# Leucémie aiguë myéloblastique et translocation (8;16) (p11;p13), premier cas marocain d'une entité clinico- biologique distinct

**DOI:** 10.11604/pamj.2015.21.147.6911

**Published:** 2015-06-23

**Authors:** Adiba Bakkali, Mouna Lemchaheb, Nezha Had, Hind Dehbi, Said Benchekroun, Asma Quessar

**Affiliations:** 1 Service d'Hématologie et d'Oncologie Pédiatrique, Hôpital 20 Août, Casablanca, Maroc; 2 Laboratoire Hda d'Analyses de Biologie Médicale, Casablanca, Maroc; 3 Faculté de Médecine et de Pharmacie de Casablanca, Laboratoire de Génétique et de Pathologie Moléculaire, Casablanca, Maroc

**Keywords:** LAM/M4-M5, t(8; 16)(p11; p13), érythrophagocytose, histiocytose, immunophenotypage, cytochimie, FISH, MYST3, CREBBP, coagulopathie intravasculaire disséminée, LAM/M4-M5, t(8; 16)(p11; p13), erythrophagocytosis, histiocytosis, immunophenotyping, cytochemistry, FISH, MYST3, CREBBP, Disseminated intravascular coagulopathy

## Abstract

La cytogénétique constitue un outil indispensable pour le diagnostic et le pronostic de la leucémie aigue myéloïde (LAM). La t(8;16)(p11;p13) est rare au cours de cette pathologie. Nous décrivons le cas d'une patiente de 22 ans, admise pour un syndrome d'insuffisance médullaire complet associé à une altération de l’état général. L'examen clinique initial montrait un purpura ecchymotique diffus et des adénopathies latérocérvicales centimétriques bilatérales. L'hémogramme avait montré une anémie à 7,6g /dl normochrome normocytaire, des globules blancs à 87,8×10^9^/L, 15% de polynucléaires neutrophiles, 60% de blastes, 24% de lymphocytes, 1% de Monocytes et 65×10^9^/L de plaquettes. Le myélogramme avait objectivé une LAM1. Sur l'immunophenotypage les marqueurs positifs étaient le CD33 (99%), le CD15 (73%), le CD38 (95%) et l'HLA-DR (88%), les marqueurs monocytoïdes CD14 et CD64 étaient positifs, le CD34, les marqueurs lymphopïdes, la MPO (26%) et le CD13 (2%) étaient négatifs. Le caryotype avait montré: t(8,16)(p11, p13) add16 (20/20). L'inversion du chromosome 16 recherchée par FISH était négative. Le traitement avait consisté en 2 cures d'induction et 2 cures de consolidation selon le protocole national de traitement des LAM (Cytarabine, daunorubicine, etoposide), la rémission complète avait été obtenue en fin d'induction I, maintenue 9 mois suivie d'une rechute; Vu l'absence de possibilité d'une allogreffe, un traitement palliatif a été instauré, la malade est décédée de sa maladie un mois après la rechute. Notre cas se présente comme les cas décrits dans la littérature avec des données clinico- biologiques particulières.

## Introduction

La leucémie aiguë myéloblastique est une hémopathie maligne qui constitue un groupe hétérogène d'entités variables sur le plan clinique, biologique, moléculaire et pronostic. Selon les recommandations de l'OMS la cytogénétique est devenue obligatoire pour le diagnostic de la LAM [1]. L'association LAM et t(8;16)(p11;p13) apparait comme une entité particulière [2], cette association a été décrite pour la première fois en 1987 [3], elle est rare, jusqu’à présent seulement une centaine de cas ont été rapportés [4], elle se voit aussi bien chez l'enfant que chez l'adulte avec des différences notamment sur le plan pronostic [5]. Vu que ce cas est le premier rapporté au Maroc et pour mieux caractériser cette aberration il nous a semblé intéressant de décrire cette observation.

## Patient et observation

**Figure 1:**
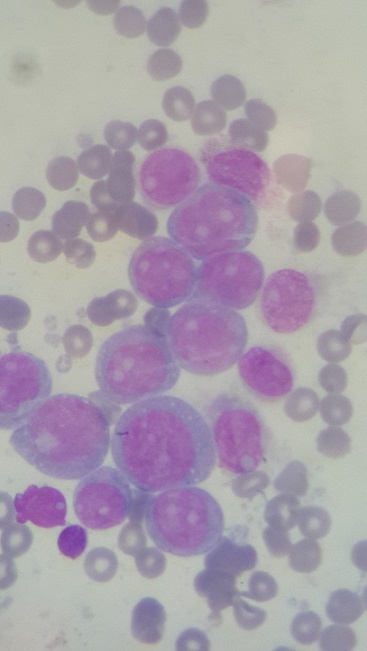
Myélogramme de notre patiente montrant une infiltration par 95% de blastes concluant à une LAM1

**Figure 2:**
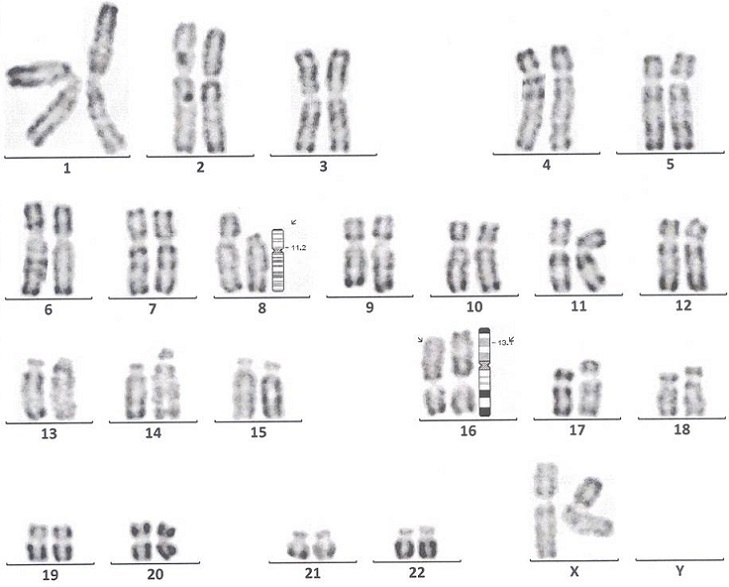
Caryotype de notre patiente montrant la translocation (8;16) (p11;p13)

Jeune patiente de 22 ans, sans antécédents pathologiques notables; admise pour un syndrome d'insuffisance médullaire complet associé à une altération profonde de l’état général installés en une semaine; L'examen physique initial montrait un performance status selon l'OMS à 3, un purpura ecchymotique diffus et des adénopathies latérocérvicales centimétriques bilatérales; L'hémogramme avait montré une anémie à 7,6 g /dl normochrome normocytaire, une hyperleucocytose à 87,8×10^9^/L, avec des polynucléaires neutrophiles à 15%, 60% blastes, 1% de Monocytes, 24% de lymphocytes et 65×10^9^//L de plaquettes;lLe Myélogramme (Figure 1) était infiltré par 95% de blastes de grande taille, granuleux à cytoplasme basophile, à noyau à chromatine fine, nucléolé, myélopéroxidase+ sur 100% des blastes (cytochimie) concluant à une LAM de type M1. En cytométrie en flux, les marqueurs positifs étaient le CD33 (99%), le CD15 (73%), le CD14 (88%) et le CD64 (100%); les blastes exprimaient HLA-DR (88%), et le CD38 (95%), le CD34, les marqueurs lymphoïdes, la MPO (26%) et le CD13 (2%) étaient négatifs. la formule du caryotype (Technique des bandes R Earle) était 46 XX, t(8,16)(p11,p16) add16 (20/20) (Figure 2); l'inversion du chromosome 16 recherchée par la technique FISH était négative. Le reste du bilan: LDH 242 UI/l, urée 0,17g /l créatinine 6mg /l, ASAT 64UI/l, ALAT 41UI/l, bilirubine 6mg /l, phosphatases alcalines 221UI/l, Gama GT 111UI/l; le Bilan d'hémostase a conclu à une coagulopathie intravasculaire disséminée (CIVD): temps de quick 12s (témoin 12s), taux de prothrombine 96%, temps de céphaline activé 25,9s (témoin 30s), fibrinogène à 1,71g/l. Le reste du bilan biologique et radiologique était sans particularités. La malade avait été mise sous protocole national de traitement des LAM « AML-MA 2011 », elle a reçu 2 cycles d'induction (induction I: Cytarabine 160mg ×2/J J1 à J10, daunorubicine 80mg J2-J4-J6; induction II: Cytarabine 160mg ×2/J J1 à J10, daunorubicine 80mg J2-J4-J6, étoposide 150mg J1 à J5) et 2 cycles de consolidation (consolidation I: Cytarabine 4,5g ×2/J J2-J3-J4, donorubicine 40mg J4 et J5, consolidation II: cytarabine 4,5g ×2/J J2-J3-J4, asparaginase 10000UI J5); La rémission complète post induction I a été maintenue moins d'une année. Après la rechute, un traitement palliatif a été proposé, vu qu'il n'y a pas de possibilité d'allogreffe; La patiente est décédée suite à la progression de sa maladie.

## Discussion

Notre patiente présentait un tableau d'insuffisance médullaire ave hyperleucocytose à 87,8×10^9^/L; Le type morphologique était M1, les marqueurs myéloïdes dont les marqueurs monocytoïdes CD14 et CD64 étaient positifs; L'issu était fatale malgré la rémission complèt. L'association LAM et t(8;16)(p11;p13) est rare, (0,2%-0,4%), [5, 6]; Notre cas est le premier constaté aussi bien chez l'enfant que chez l'adulte; Elle est caractérisée par la fréquence des localisations extra médullaires, [2] en particulier du système nerveux central, une hépatomégalie, une splénomégalie et des lymphadénopathies sont aussi fréquentes [4, 5]; Sur le plan biologique, elle est souvent associée à une perturbation du bilan d'hémostase, en particulier à une CIVD [2]. Le sous type morphologique est souvent M4 et M5 [6], certaines études décrivent la classification cytomorphologique difficile chez l'adulte [4], en effet l'origine à partir d'une cellule souche à potentiel myéloïde et monoblastique est suggéré comme hypothèse étiopathogénique [4]. Les noyaux monocytoïdes sont vus dans la LAM3 hypogranulaire, et les blastes peuvent être hypogranulaires le diagnostic différentiel se pose alors avec la LAM promyélocytaire [2], un autre diagnostic différentiel est l'histiocytose devant l'erythrophagocytose [2] souvent constatée [6]. Cette entité a pu être mieux identifiée par la biologie moléculaire qui a mis en évidence le réarrangement qui fusionne le gène MOZ (monocytic leukemia zinc finger) /MYST3 au niveau de la bande 8p11 avec le gène CBP (CREB binding protein)/CREBBP au niveau de la bande 16p13 (codant tous les deux pour une acétyl transférase) [2, 6, 7]. Concernant le pronostic, la survie globale moyenne chez l'adulte est de 4,7 mois, chez l'enfant la survie à5 ans est de 59%, des rémissions complètes spontanées ont été observées chez le nouveau né [5]. Le Tableau 1 résume les caractéristiques de cette anomalie en fonction de l’âge.

**Tableau 1:** Particularités de la t(8;16)(p11;p13) en fonction de l’âge [4, 5]

	Adultes	Enfants
**Incidence**	Rare (0,2%); 70%: femmes	Rare: < 100 cas décrits (enfants + adultes)
**Présentation clinique**	- Forme post thérapeutique fréquente - Hépatomégalie (41%) splénomégalie (33%) - Adénopathies (37%)	- Coagolopathie intravasculaire disséminée (39%) - Atteinte extra médullaire: 66% (système nerveux central, sarcome granulocytaire
**Cytologie**	- MPO et estérases non spécifiques + - Classification franco-américano-britanique Impossible - Erythrophagocytose	- LAM4 et LAM5 (97%) - Erythrophagocytose (70%)
**Cytométrie en flux**	- CD56 (71% des cas) - MOP+, CD33+, CD13+, CD65+, CD15+, CD34-, CD117-, CD113-, CD14+, CD64+, CD11b+, CD4+	- Marqueurs myéloïdes positifs: MPO, D13, CD33 -CD15+, HLA-DR+ -CD14+
**Biologie moléculaire**	- Le gène MOZ/MYST3 au niveau de la bande 8p11 fusionne au niveau 16p13avec le gène CBP/CREBBP - Surexpression des gènes HOXA, RET, PRL, CHD3, CPEB2, NR2F6	Les gènes hautement exprimés: - HOXA11 - HOXA10 - RET - PERP - GGA2
**Pronostic**	- Très mauvais - Survie globale moyenne 4,7 mois	- Contrairement à l'adulte évolution comme LAM sans t(8;16) - Survie à 5 ans 59% - Rémission spontanée possible chez les nouveau-nés

## Conclusion

L'association t(8;16) (p11;p13) et LAM est rare. Dans notre cas bien que le type morphologique était M1 les marqueurs monocytoïdes étaient positifs. Les limites de cette étude c'est le manque de la biologie moléculaire qui a un rôle pronostic.

## References

[1] Dohner H, Estey E, Amadori S (2010). Diagnosis and managment of acute myeloid leukemia in adults: recommendations from an international expert panel, on behalf of the European leukemiaNet. Blood..

[2] Tsieh S, Ernest W (2001). Acute Monoblastic Leukemia With t(8;16):a Distinct Clinicopathologic Entity; Report of a Case and Review of the Literature. Am J Hematol..

[3] Heim S, Avanzi GC, Billstrom R (1987). A new specific chromosomal rearrangement t(8,16)(p11,p13) in acute monocytic leukemia. Br J Haematol..

[4] Haferlach T, Kohlmann A, Klein H (2009). AML with translocation t(8;16)(p11;p13) demonstrates unique cytomorphological, cytogenetic, molecular and prognostic features. Leukemia..

[5] Eva A, Coenen C, Michel Z (2013). Pediatric acute myeloid leukemia with t(8;16)(p11;p13), a distinct clinical and biological entity: a collaborative study by the International-Berlin-Frankfurt-Münster AML-study group. Blood..

[6] Paolo B, Ester O, Paola C (2000). Translocation (8;16) in a patient with acute myelomonocytic leukemia occurring after treatment with fludarabine for a low-grade non-Hodgkin's lymphoma. Haematologica..

[7] Murati A, Adélaïde J, Quilichini B (2007). New types of MYST3-CBP and CBP-MYST3 fusion transcripts in t(8;16)(p11;p13) acute myeloid Leukemias. Haematologica..

